# Outcomes and Risk Factors of Patients With COVID-19 and Cancer (ONCORONA): Findings from The Philippine CORONA Study

**DOI:** 10.3389/fonc.2022.857076

**Published:** 2022-04-13

**Authors:** Adrian I. Espiritu, Ramon B. Larrazabal, Marie Charmaine C. Sy, Emilio Q. Villanueva, Veeda Michelle M. Anlacan, Roland Dominic G. Jamora

**Affiliations:** ^1^ Department of Neurosciences, College of Medicine and Philippine General Hospital, University of the Philippines Manila, Manila, Philippines; ^2^ Department of Clinical Epidemiology, College of Medicine, University of the Philippines Manila, Manila, Philippines; ^3^ Division of Neurology, Department of Medicine, St. Michael’s Hospital, University of Toronto, Toronto, ON, Canada; ^4^ Division of Medical Oncology, Department of Medicine, College of Medicine and Philippine General Hospital, University of the Philippines Manila, Manila, Philippines; ^5^ Department of Pathology, College of Medicine and Philippine General Hospital, University of the Philippines Manila, Manila, Philippines; ^6^ Institute for Neurosciences, St. Luke’s Medical Center, Taguig, Philippines

**Keywords:** cancer, mortality, respiratory failure, intensive care unit admission, outcomes, COVID-19

## Abstract

**Background:**

A study conducted in China on patients with coronavirus disease 2019 (COVID-19) showed that cancer conferred a five times increased risk for needing intensive care admission and mortality; No data has yet been collected and published from the Philippines. Thus, the investigators conducted this substudy to determine the association of having a history of cancer with clinical outcomes among patients included in the Philippine CORONA Study.

**Methodology:**

Multi-center, retrospective cohort design

**Results:**

A total of 244 patients had a history of cancer, out of 10,881 COVID-19 hospital admissions. After adjusting for different confounding variables of interest, having cancer was significantly associated with a 75% increased odds of having severe/critical COVID-19 at nadir (CI 95% 1.32, 2.33; *p* < 0.001). After adjusting for different confounding variables of interest, having cancer was significantly associated with the following time-to-event outcomes: 72% increase in hazard of in-hospital mortality (CI 95% 1.37, 2.16; *p* < 0.001), 65% increase in hazard of respiratory failure (CI 95% 1.31, 2.08; *p* < 0.001), and 57% increase in hazard of being admitted to ICU (CI 95% 1.24, 1.97; *p* < 0.001).

**Conclusion:**

A history of cancer conferred poorer clinical outcomes on adult, hospitalized COVID-19 patients.

## Introduction

On the 11^th^ of March 2020, the World Health Organization declared that the coronavirus disease 2019 (COVID-19), caused by the severe acute respiratory syndrome coronavirus 2 (SARS-CoV-2), was a global pandemic (1). Since then, it has affected more than 420 million people, causing 5.86 million deaths, as of February 19, 2022 worldwide (2). At the same time in the Philippines, this pandemic has affected an estimated 3.65 million people with more than 55 thousand deaths (3).

Studies have been conducted worldwide that evaluated the demographic profiles, clinical characteristics and clinical outcomes among COVID-19 patients. There are other specific subgroups, such as those with cancer, that are susceptible to the disease and are associated with poorer clinical outcomes such as requiring oxygen support, needing intensive care, and increased mortality (4). A study conducted in Italy supported this when they showed that cancer conferred a near-doubled death rate among patients with cancer than those without who were infected with COVID 19 (5). Furthermore, the published data from the COVID-19 in patients with thoracic malignancies study and clinical impact of COVID-19 patients with cancer study, both being registries from different countries, showed that age, smoking, male sex, functional status, and the presence of active cancer were associated with higher mortality. Specifically, the COVID-19 study done by the Gustave Roussy Hospital identified that those older than 70 years old, with a smoking history, advanced cancer (metastatic), those undergoing cytotoxic chemotherapy, and those having an Eastern Cooperative Oncology Group performance status of more than or equal to 2 are risk factors that increased the risk of death (6).

The studies mentioned were conducted in Europe, South America, United States of America, Japan, and China; and no data was collected and published from the Philippines, a developing country with a different healthcare system (7). Thus, the investigators in collaboration with the Philippine CORONA Study Group conducted a substudy on the cohort of COVID-19 patients with a history of cancer; looking at their clinical outcomes (i.e., mortality, respiratory failure, duration of ventilator dependence, intensive care unit (ICU) admission, length of ICU and hospital stay compared to those with no history of cancer (8).

## Materials and Methods

### Study Design

This was a retrospective substudy of patients with cancer enrolled in the Philippine CORONA study, which was a nationwide, multicentre, comparative, retrospective, cohort study of hospitalized patients with COVID-19 from February 2020 to December 2020. The study protocol was registered in ClinicalTrials.gov (NCT04386083) and published previously (8).

### Study Setting

This substudy was based on the data gathered from 37 major hospitals/study sites from various regions in the Philippines. The specific sites are listed in the published study protocol (8).

### Patient Selection

We included a total enumeration of all patients that fulfilled the inclusion criteria, as follows: adults older than or of 19 years of age; confirmed cases *via* COVID-19 real-time reverse transcription polymerase chain reaction (RT-PCR) of patients’ nasopharyngeal swab samples which were performed by testing centers accredited by the Department of Health (Philippines); clinical symptoms/signs ascribed to COVID-19 infection; patients with the appropriate disposition by the end of the data collection period (e.g., discharged, transferred to another hospital, or died). Individuals who were transferred to another hospital were excluded to prevent duplication of data. Adult COVID-19 patients who had new-onset neurological symptom/s (NNS) were grouped under the exposed cohort while those without NNS (non-NNS) were classified under the unexposed cohort. The information on the patient selection is detailed in the published study protocol (8).

### Data Collection Procedure and Management

All investigators, sub-investigators, and hired data collectors obtained pertinent information for this study. An electronic data collection form was formulated using Epi Info™ Software (Version 7.2.2.16). The form was pilot-tested and a formal orientation and workshop was conducted to ensure accurate data collection as explained in the published protocol (8). Moreover, the data collection forms used did not contain any information that could identify the patients.

The following pertinent data were obtained: demographic data; other clinical profile data/comorbidities; neurological history; date of illness onset; respiratory and constitutional symptoms associated with COVID-19; COVID-19 disease severity25 at nadir; data if neurological manifestation/s were present at onset prior to respiratory symptoms and the specific neurological manifestation/s present at onset; neurological symptoms; date of neurological symptom onset; new-onset neurological disorders or complications; date of new neurological disorder or complication onset; imaging done; cerebrospinal fluid analysis; electrophysiological studies; treatment given; antibiotics given; neurological interventions given; date of mortality and cause/s of mortality; date of respiratory failure onset, date of mechanical ventilator cessation and cause/s of respiratory failure; date of first day of ICU admission, date of discharge from ICU and indication/s for ICU admission; other neurological outcomes at discharge; date of hospital discharge; and final disposition.

### Statistical Analysis

Baseline characteristics and clinical outcomes of the patients were summarized by descriptive statistics. Numerical variables were described as mean and standard deviation, if the data were normally distributed as assessed by the Shapiro-Wilk test for normality, and as median and interquartile range, if otherwise. Categorical variables were described as frequency and proportion. These different baseline characteristics and clinical outcomes were compared between the two groups: with cancer, and without cancer. A significant difference in the mean/median/mean-rank of the different numerical variables between the two groups was determined by Student’s t test for the variables with normally distributed data, while Mann-Whitney U test was done for non-normally distributed variables. Comparison of the proportions of the different categorical variables between the two groups was determined by chi-squared test or Fisher exact test.

The associations between having cancer and the different individual dichotomous outcome variables of interest were determined by multivariable binary logistic regression. Survival analysis was also done for time-to-event data of mortality, respiratory failure, and admission to ICU. The time-to-event were right-censored on time-to-discharge as the exit from the time-at-risk among those who have not experienced the event, i.e., mortality, respiratory failure, or admission to ICU, during the hospital stay. The associations between having cancer and the different time-to-event outcome variables of interest were determined by multivariable Cox proportional hazards regression. The logistic and Cox proportional hazards regression models were adjusted for the following pre-determined confounders: age group, sex, smoking status, hypertension, chronic cardiac disease, chronic respiratory disease, chronic kidney disease, and chronic neurologic disease. A cutoff of *p* < 0.05 identifies having cancer as a significant predictor of the different outcomes of interest. Kaplan-Meier curves were constructed to visualize the survival curves of those with cancer versus without cancer; adjusted for the different confounding variables of interest, and also for the different time-to-event outcome variables.

## Results

**Figure 1:**
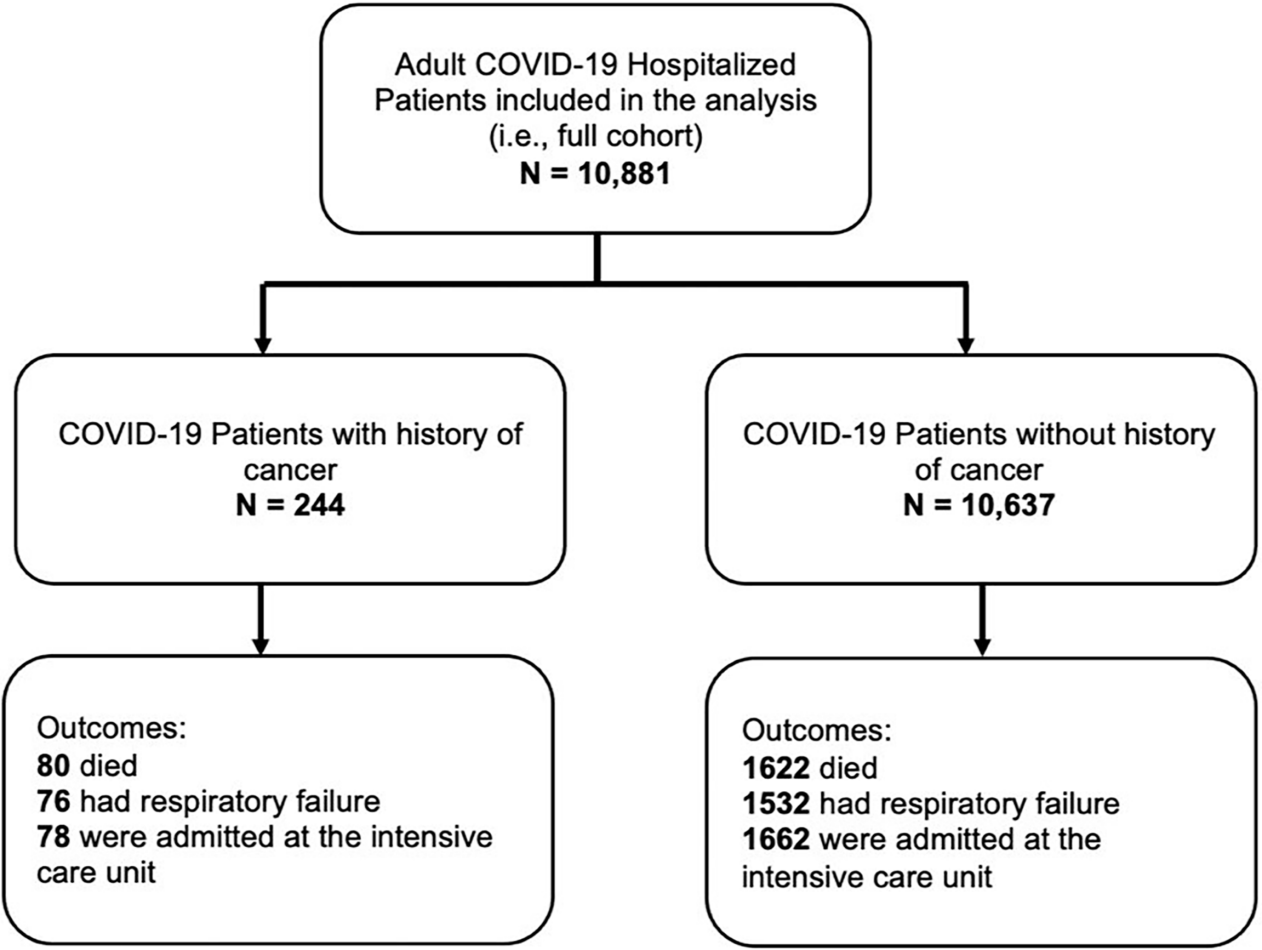
Flow of patients.

A total of 10,999 patients were hospitalized who tested positive for COVID 19 (by reverse transcription polymerase chain reaction) from February to December 2020 were included in the study. Initially, there were 118 patients who did not meet the age criteria, and were excluded in the final analysis in the published paper (9). Out of the 10,881 patients included in the final analysis, 244 of them had a history of cancer (**see** **Figure 1**).

### Demographic and Clinical Profile

The participants’ age were divided into 2 categories: those aged 60 years and above and those aged 18-59 years. The cancer group had equal participants for both age groups [122 (50%) vs 122 (50%)], while majority of the group without cancer were belonged to the less than 60 years old age group [6925 (65.1%) vs 3,712 (34.9%), *p* < 0.001]. There was a considerable number of women in the study and significantly more reported having no history of cancer [4950 (46.5%), *p* < 0.001].

The most common non-neurologic co-morbidity reported was hypertension in both groups with and without cancer. There was a significant difference between groups, with those with a history of cancer reporting to have more hypertension [113 (46.3%) vs 3534 (33.2%), *p* < 0.001]. The most common neurologic co-morbidity reported was a history of stroke which was significantly different between both groups [14 (5.7%) vs 307 (2.9%), *p* 0.009], higher in those with a history of cancer than those without.

The group of patients with cancer (or a history of cancer) was noted to have a significantly higher incidence of respiratory symptoms: cough [128 (52.5%) vs 4,283 (40.3%), *p* < 0.001], dyspnea [98 (40.2%) vs 2605 (24.5%), *p* < 0.001), and increased sputum production [28 (11.5%) vs 609 (5.7%), *p* < 0.001]. It also showed that the cancer group was given more glucocorticoids [93 (38.1%) vs 2751 (25.9%), *p* < 0.001], antiviral medications [60 (24.6%) vs 1842 (17.3%)], and antibiotics [221 (90.6%) vs 8793 (82.7%), *p* < 0.001] as treatment for COVID 19, compared to those patients without a history of cancer. The cancer group experienced more symptoms and needed more medications (**see** **Table 1**).

**Table 1:** Clinicodemographic characteristics of the stratified according to having cancer or without.

	With cancer (n = 244)	Without cancer (n = 10,637)	p-value
**Socio-demographic data**			
Age group			<0.001
19 – 59 y, n (%)	122 (50.0)	6925 (65.1)	
≥ 60 y, n (%)	122 (50.0)	3712 (34.9)	
Female, n (%)	149 (61.1)	4950 (46.5)	<0.001
Ever-smoker (past/current), n (%)	31 (12.7)	995 (9.4)	<0.001
**Non-neurologic comorbidities, n (%)**			
Hypertension	113 (46.3)	3534 (33.2)	<0.001
Diabetes mellitus	64 (26.2)	2127 (20.0)	0.016
Chronic cardiac disease^a^	20 (8.2)	492 (4.6)	0.009
Chronic respiratory disease^b^	12 (4.9)	604 (5.7)	0.611
Chronic kidney disease	24 (9.8)	587 (5.5)	0.004
Chronic liver disease	1 (0.4)	59 (0.6)	1.000
HIV/AIDS	1 (0.4)	36 (0.3)	0.569
**Past neurologic history, n (%)**			
Stroke/cerebrovascular	14 (5.7)	307 (2.9)	0.009
Epilepsy	1 (0.4)	26 (0.2)	0.458
Neurodegenerative^c^	3 (1.2)	41 (0.4)	0.075
Headache syndrome	-	5 (0.05)	1.000
Demyelinating disorder	-	2 (0.02)	1.000
Central nervous system infection	-	5 (0.05)	1.000
Peripheral nervous system disorders^d^	-	15 (0.1)	1.000
**Respiratory and constitutional symptoms, n (%)**			
Fever	107 (43.8)	3820 (35.9)	0.011
Cough	128 (52.5)	4283 (40.3)	<0.001
Dyspnea	98 (40.2)	2605 (24.5)	<0.001
Rhinorrhea	9 (2.7)	598 (5.6)	0.193
Sputum production	28 (11.5)	609 (5.7)	<0.001
Sore throat	15 (6.2)	736 (6.9)	0.638
Diarrhea	19 (7.8)	578 (5.4)	0.111
Fatigue	16 (6.6)	697 (6.6)	0.998
Others	53 (21.7)	1621 (15.2)	0.006
**New onset neurological symptoms, n (%)**			
Headache	10 (4.1)	597 (5.6)	0.308
Nausea or vomiting	8 (3.3)	150 (1.4)	0.026
Seizure	5 (2.0)	91 (0.9)	0.064
Altered mental state^e^	28 (11.5)	490 (4.6)	<0.001
Olfactory or taste dysfunction	14 (5.7)	649 (6.1)	0.814
Dysfunctions of other senses^f^	9 (3.7)	157 (1.5)	0.012
Bulbar symptoms^g^	3 (1.2)	119 (1.1)	0.755
Motor symptoms	13 (5.3)	233 (2.2)	0.001
Sensory symptoms	4 (1.6)	49 (0.5)	0.031
Myalgia	5 (2.0)	251 (2.4)	0.752
Others^h^	-	33 (0.3)	1.000
**New-onset neurological disorders/complications, n (%)**			
Encephalopathy^i^	35 (14.3)	609 (5.7)	<0.001
Symptomatic seizure/status epilepticus	7 (2.9)	118 (1.1)	0.022
Stroke/cerebrovascular^i^	13 (5.3)	354 (3.3)	0.087
Central nervous system infection^k^	–	7 (0.1)	1.000
Others^l^	–	14 (0.1)	1.000
**Treatment/s received, n (%)**			
Glucocorticoids	93 (38.1)	2751 (25.9)	<0.001
Tocilizumab	27 (11.1)	1002 (9.4)	0.385
Antiviral^m^	60 (24.6)	1842 (17.3)	0.003
Antibacterial	221 (90.6)	8793 (82.7)	0.001
Others^n^	96 (39.3)	3809 (35.9)	0.255

a Includes heart failure, coronary artery disease, prior history of myocardial infarction, and other cardiac conditions.

b Includes bronchial asthma. chronic obstructive pulmonary disease, restrictive lung disease, and other pulmonary conditions.

c Includes dementia and movement disorders

d Includes peripheral nervous system infection, peripheral nerve disease, neuromuscular junction disorder, and muscle disorder.

e Includes altered sensorium and confusion.

f Includes visual, hearing, and vestibular dysfunctions.

g Includes facial paresthesia, facial weakness, dysarthria, dysphonia, dysphagia, tongue weakness, and neck weakness.

h Includes tremor, dystonia, choreoathetosis, bradykinesia, ataxia, and meningisimus.

i Includes encephalopathy, and anoxic brain injury.

j Any acute cerebrovascular disease (no need to distinguish between cerebrovascular disease infarction, hemorrhagic).

k Includes encephalitis, meningitis, and meningoencephalitis.

l Includes acute disseminated encephalopmyelitis, optic neuritis, sensory ganglionitis, radiculitis, anterior horn syndrome, peripheral neuritis (Guillain-Barre Syndrome [GBS], other than GBS), neuromuscular disorder, and myositis.

m Includes remdesivir, lopinavir, and ritonavir.

n Includes chloroquine, hydroxychloroquine, convalescent plasma, and other therapies.

### Comparison of Outcomes of the Patients According to Whether They Have Cancer or Not

Our study showed that there was significantly higher proportion of patients with cancer with the following outcomes of interest than those patients without cancer: severe/critical COVID-19 at nadir [125 (52.1%) vs 3936 (37.5%), *p* < 0.001], in-hospital mortality [80 (32.8%) vs 1622 (15.3%), *p* < 0.001], respiratory failure [76 (31.1%) vs 1532 (14.4%), *p* < 0.001], intensive care unit (ICU) admission [78 (31.9%) vs 1662 (15.6%), *p* < 0.001], hospital stay > 14 days [113 (46.3%) vs 4191 (39.4%), *p* 0.026], and neurologic presentation or complication [80 (32.8%) vs 2211 (20.8%), *p* < 0.001] (**see** **Table 2**).

**Table 2:** Clinical outcomes of COVID-19 patients stratified according to having cancer or without.

Outcomes	With cancer	Without cancer	p-value
	(n = 244)	(n = 10,637)	
**COVID-19 severity at nadir**			<0.001
Mild/moderate, n (%)	115 (47.9)	6575 (62.6%)	
Severe/critical, n (%)	125 (52.1)	3936 (37.4%)	
**In-hospital mortality,** n (%)	80 (32.8)	1622 (15.2%)	<0.001
Time to in-hospital mortality in days, median (IQR)	15 (9)	15 (14)	0.613
**Respiratory failure, n (%)**	76 (31.2)	1532 (14.4)	<0.001
Time to respiratory failure in days, median (IQR)	5 (4)	5 (4)	0.815
Duration of IMV in days, median (IQR)	13 (10.5)	13 (12)	0.899
IMV dependence ≤ 5 days, n (%)	10 (4.1)	208 (2)	0.032
IMV dependence > 5 days, n (%)	234 (95.9)	10,429 (98)	
**Admitted to ICU, n (%)**	78 (32)	1662 (15.6)	<0.001
Time to ICU admission in days, median (IQR)	4.5 (3)	5 (4)	0.721
Length of ICU stay in days, median (IQR)	15 (10)	15 (12)	0.996
ICU stay ≤ 7 days, n (%)	9 (11.5)	263 (15.8)	0.308
ICU stay > 7 days, n (%)	69 (88.5)	1399 (84.2)	
**Length of hospital stay** ^a^ **in days, median (IQR)**	14 (10)	13 (9)	0.026
Hospital stay ≤ 14 days, n (%)	131 (53.7)	6446 (60.6)	0.029
Hospital stay > 14 days, n (%)	113 (46.3)	4191 (39.4)	
**Neurologic presentation or complication, n (%)**	80 (32.8)	2211 (20.8)	<0.001
Neurologic outcome^b^			0.006
Full/partial neurologic recovery, n (%)	34 (72.3)	1605 (86.4)	
No recovery, n (%)	13 (27.7)	253 (13.6)	

ICU, intensive care unit; IMV, invasive mechanical ventilation; IQR, interquartile range.

a Derived from overall length of stay for patients who were never admitted to ICU; excludes length of ICU stay for those who were admitted in the ICU.

b Patients with recorded data for neurologic outcome (n=1905).

Additionally, there were significantly lower proportion of patients with cancer with the following outcomes of interest than those patients without cancer: IMV dependence >5 days [234 (95.9%) vs 10,429 (98%), *p* < 0.032] and full/partial neurologic recovery among patients with neurologic presentation at admission or neurologic complication during hospital stay [34 (72.3%) vs 1605 (86.3%), *p* < 0.001].

### The Relationship of Clinical Outcomes of Interest Among Patients With and Without Cancer

After adjusting for the different confounding variables of interest, having cancer was significantly associated with the following outcomes: those with cancer have 75% increased odds of having severe/critical COVID-19 at nadir [OR 1.75, 95% CI 1.32, 2.33], a 54% increased odds of having neurologic presentations/complications [OR 1.54, 95% CI 1.17, 2.03], and a 54% decreased odds of having full/partial neurological improvement if they have neurologic presentations/complications [OR 0.46, 95% CI 0.22, 0.93] (**see** **Table 3**).

**Table 3:** Association of having a cancer with the different outcomes of interest.

Outcomes*	Crude OR	95% CI	Adjusted OR*	95% CI
Severe/critical COVID-19 at nadir	1.82	1.41, 2.35	1.75	1.32, 2.33
Neurological presentation/complication	1.86	1.42, 2.44	1.54	1.17, 2.03
Full/partial neurological improvement	0.41	0.21, 0.79	0.46	0.22, 0.93
IMV dependence > 5 days	0.47	0.24, 0.89	0.58	0.3, 1.13
ICU stay > 7 days	1.44	0.71, 2.92	1.39	0.68, 2.83
Hospital stay > 14 days	1.33	1.03, 1.71	1.22	0.94, 1.58

IMV, invasive mechanical ventilation; ICU, intensive care unit.

*Individual univariate multiple logistic regression analysis with independent variable malignancy adjusted for age group, sex, smoking history, hypertension, diabetes, chronic cardiac disease, chronic kidney disease, chronic respiratory disease, and chronic neurologic disease.

**Figure 2:**
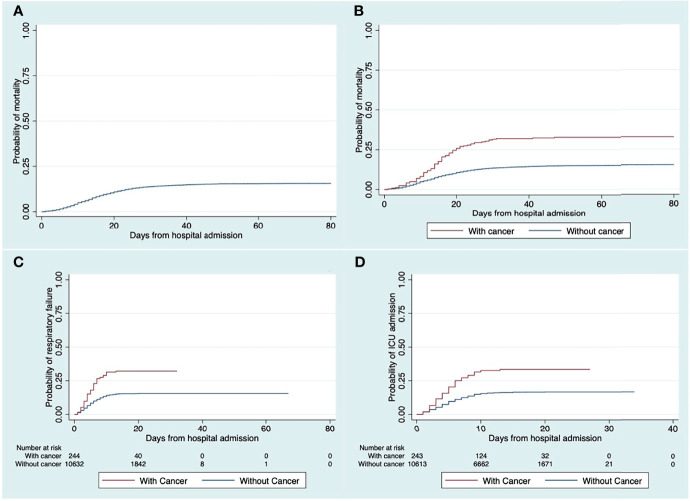
Kaplan-Meier failure plot of the full cohort **(A)**, and the comparison of the probability of **(B)** mortality, **(C)** respiratory failure, and **(D)** ICU admission, between COVID-19 patients with and without cancer.

After adjusting for the different confounding variables of interest, having cancer was significantly associated with the following time-to-event outcomes (**see** **Table 4**): 72% increase in hazard of in-hospital mortality [HR 1.72, 95% CI 1.37, 2.16], 65% increase in hazard of respiratory failure [HR 1.65, 95% CI 1.31, 2.08], and a 57% increase in hazard of being admitted to ICU [HR 1.57, 95% CI 1.24, 1.97]. The following outcomes are depicted in the Kaplan-Meier curves (**see** **Figure 2**).

**Table 4:** Association of having cancer with the different outcomes of interest (time-to-event analysis).

Outcomes*	Crude HR	95% CI	Adjusted HR*	95% CI
In-hospital mortality	2.07	1.65, 2.59	1.72	1.37, 2.16
Respiratory failure	2.33	1.85, 2.94	1.65	1.31, 2.08
ICU admission	2.25	1.79, 2.83	1.57	1.24, 1.97

ICU, intensive care unit.

*Individual univariate multiple Cox proportional hazard regression analysis with independent variable malignancy adjusted for age group, sex, smoking history, hypertension, diabetes, chronic cardiac disease, chronic kidney disease, chronic respiratory disease, and chronic neurologic disease.

## Discussion

Previous literature have shown that patients with cancer are as susceptible to being infected with the SARS-CoV-2 virus as those without (10, 11). This study emphasized that the cohort of COVID-19 patients with cancer had a higher risk for in-hospital mortality, respiratory failure, and needing intensive care. This was consistent with early studies reported in China (4–7). There was a 72% increase in in-hospital mortality was in stark contrast to the other studies that analyzed a similar cohort of patients done in New York City (12, 13). The investigators attribute this high mortality in our study to a difference in healthcare systems. In the Philippines the universal health care law is partially and poorly implemented (14) and majority of the Filipinos belong to the lower socioeconomic status (15). Since 1992, the Philippine Government has devolved the management and delivery of health services from the national Department of Health to locally elected provincial, city, and municipal governments. This was highlighted in a review of studies on the health care cost and financing of patients with malignancies (especially for those with central nervous system malignancies). The cost of care (especially in cancer care) in the country is not covered by most insurance companies and is scarcely subsidized by the state, which means that cancer care is an out-of-pocket cost for most if not all Filipinos (14, 15).

Our cohort also analyzed multiple centers nationwide, with varying levels of healthcare services, and not just a single-center study (9). There was also the consideration of poorer socioeconomic factors (residence in rural areas and low level of income) which are associated with increased mortality (16). The other outcomes of interest in this study were consistent with other studies such as respiratory failure and needing intensive care which, along with increased mortality, all portend to poor clinical outcomes shown in patients with cancer (6, 7).

In response to the surge of cases in the country, the Philippine General Hospital (PGH) was designated by the Philippine Department of Health as a COVID-19 referral center. The Department of Health also designated other institutions in different regions as COVID-19 referral centers. This meant that since March 31, 2020 non-emergency clinics, elective procedures and surgeries and non-COVID-19 admissions were intermittently being suspended or limited depending on the threat of the COVID surge. Realizing the possible detrimental effects of the cessation of oncologic care, the Cancer Institute adapted strategies that allowed the continuation of cancer care and maintained the safety and well-being of both the patients and the health professionals. Patients seen at the clinic were limited and appointments given were based on a prioritization scheme. The Philippine government has relegated the duty of vaccinating individuals to the local governments. As of this writing, 62.2 million people (56.8%) have been fully vaccinated (2).

The pandemic has a profound impact on cancer care in all aspects. Specifically, the screening, diagnosis, and treatment in both medical and surgical oncology. With the participants recruited many months into the pandemic, those patients with cancer might have had their treatment delayed due to the burden the pandemic imposed on the healthcare system (i.e. prioritizing patients with COVID-19) as well as the government response (i.e. logistics problem due to the numerous and repeated lockdowns imposed) (16). With these roadblocks to cancer care, an international collaborative group recommended a prioritization scheme that can maximize health benefits, taking into consideration the patient, their disease, and prognosis (17).

In our study, we were able to show a significant difference of neurologic manifestations between patients with and without cancer. Only altered mental status was shown to be more in those with cancer. The presence of new-onset neurologic manifestations could be explained by the COVID-19 infection itself, as reported in a meta-analysis showing that COVID 19 may manifest primarily or initially with neurologic manifestations (18). This neurologic involvement was also seen neuropathologically, where direct central nervous system involvement was documented (19). Although the direct relationship between COVID 19 and its effect on the nervous system is yet to be fully established, it has been postulated that there is the presence of severe hemorrhage and hypoxia, increased thrombotic events (as seen in infections like COVID 19), acute disseminated encephalomyelitis – like changes, encephalitis, and meningitis. Lastly, there have been reports of SARS-CoV-2 (the causative agent of COVID 19) reactivity in the brain (19). While COVID-19 patients present with abnormal electroencephalograms, these are not specific for COVID 19 alone and may be attributed to COVID-19 causing inflammation in the brain or other extracranial causes of encephalopathy (20).

A study done in Europe analyzed 1044 patients with active cancer diagnosed with COVID 19 and their outcomes, around 30.6% patients in the study died, while 92.5% of whom had a cause of death recorded as due to COVID-19. Furthermore, the all-cause case–fatality rate in patients with cancer who were infected with COVID-19 was significantly associated with sex and older age (21). A meta-analysis done in China included 15 studies from different countries around the world showed that the overall case fatality rate of COVID 19 patient with cancer was 22.4% (22). The relationship between cancer an COVID 19 was further established by another study which analyzed patients with cancer who were recently and had infection with COVID 19. It showed cancer patients who underwent recent chemotherapy and were associated with worse outcomes. This is explained by the possible immunosuppressive effects of chemotherapy (4).

The other effects of the pandemic on patients with cancer include tumor stage migration and higher early mortality which are both directly and indirectly caused. A study showed that there were more patients seen during the pandemic that presented to have inoperable or metastatic cancer (49.8% vs 39%). This meant poorer prognosis and that these patients most likely would only warrant palliative care (chemotherapy and/or radiotherapy) instead of being seen at an earlier stage or less extensive disease in which curative treatment (surgery, chemotherapy, and/or radiotherapy) maybe be offered (23). The 90-day mortality after the diagnosis of cancer was also higher during the pandemic at 10.5% vs 6.6%. The reason cited in the study was the higher rate of advanced disease at first referral/diagnosis which was attributed to the indirect effect of the pandemic from the lockdowns and logistical limitations for patients to seek consult and have work-up done (23). Another study supported the findings of the study cited earlier wherein, as compared to the pre-pandemic times, the diagnosis of the six most common types of cancers were lesser (24). It does not mean that the actual incidence of cancer has decreased but most likely, the ability of patients to seek consult and the facilities to conduct the work-up for the diagnosis of cancer have been hampered due to the logistic limitations caused by the pandemic.

The results of this study allowed us to understand the consequences of COVID-19 on adult patients with cancer. First, mortality rate of adult hospitalized cancer patients with COVID-19 was high, with estimates at around 32-33%. Second, cancer was also associated with longer hospital stays, respiratory failure, and needing ICU admission. Third, COVID-19 patients with cancer needed more interventions like steroids, antivirals, and antibiotics. These pieces of information may provide context and ideas for other investigators to write and conduct prospective studies and/or randomized clinical trials for this specific vulnerable subgroup.

The investigators have identified several limitations of this study. First, a detailed history on the cancer of the participants could have given us more insight on other relevant variables and their relationships; Since poorer functional status and those presently receiving cytotoxic chemotherapy had poorer outcomes compared to those who were deemed to have been cured of their cancer, with the latter having comparable outcomes to those with no cancer (25). Second, only the history of cancer was noted; with no data on the type of cancer, site, stage, and treatment history. The retrospective nature of the study was also subject to recall and selection bias. With the limitations identified, we recommend that a more detailed cancer history be taken with a prospective study design to corroborate our conclusions.

The findings in this study raise the need to further investigate patients with cancer, focusing on the type and stage of their cancer, as well as the status of their needs and treatment in relation to the pandemic. The data also stresses the importance of giving more support to vulnerable groups like patients with cancer during this time of the pandemic, in order to improve their outcomes. Furthermore, this study adds to the body of knowledge regarding COVID 19 and cancer. Physicians will be able to prognosticate this cohort of patients and government agencies in charge of the pandemic response will be able to appropriately categorize patients with cancer as a priority for vaccinations and should also be given exceptions to logistic restrictions when seeking treatment.

## Conclusion

A history of cancer conferred poorer clinical outcomes on adult, hospitalized patients with COVID-19 with increased mortality, respiratory failure, and need for ICU admission. Other demographic and clinical risk factors associated with cancer patients with COVID-19 were older age, female sex, multiple co-morbidities, and having more respiratory symptoms and neurologic manifestations.

## Data Availability Statement

The raw data supporting the conclusions of this article will be made available by the authors, without undue reservation.

## Author Contributions

AE: Conceptualization, data curation, formal analysis, interpretation of data, writing-original draft, writing-review, and editing. RL: Data curation, formal analysis, interpretation of data, writing-original draft, writing-review, and editing. MS: Conceptualization, data curation, formal analysis, interpretation of data, writing-review, and editing. EV: Data curation, formal analysis, interpretation of data, and editing. VA: Conceptualization, data curation, formal analysis, interpretation of data, writing-review, and editing. RJ: Conceptualization, data curation, formal analysis, interpretation of data, writing-original draft, writing-review, and editing. All authors contributed to the article and approved the submitted version.

## Conflict of Interest

The authors declare that the research was conducted in the absence of any commercial or financial relationships that could be construed as a potential conflict of interest.

## Publisher’s Note

All claims expressed in this article are solely those of the authors and do not necessarily represent those of their affiliated organizations, or those of the publisher, the editors and the reviewers. Any product that may be evaluated in this article, or claim that may be made by its manufacturer, is not guaranteed or endorsed by the publisher.
